# Retraction: Combinatorial approach of *in silico* and *in vitro* evaluation of MLH1 variant associated with lynch syndrome like metastatic colorectal cancer

**DOI:** 10.1042/BSR-20200225_RET

**Published:** 2020-07-02

**Keywords:** Colorectal cancer, In silico tools, In vitro analysis, Lynch syndrome, MLH1, WES

This Retraction follows an Expression of Concern relating to this article previously published by Portland Press.

This article is being retracted from *Bioscience Reports* at the request of the authors following receipt of a notification from a reader, alerting the authors to duplications in panels of Figure 3A.

The authors are unable to correct the article and wish to retract the article. The Editor-in-Chief and Editorial Board agree with the retraction.

